# Synthesis and Applications of Dimensional SnS_2_ and SnS_2_/Carbon Nanomaterials

**DOI:** 10.3390/nano12244497

**Published:** 2022-12-19

**Authors:** Catherine Sekyerebea Diko, Maurice Abitonze, Yining Liu, Yimin Zhu, Yan Yang

**Affiliations:** 1 College of Environmental Science and Engineering, Dalian Maritime University, Dalian 116026, China; 2 Dalian Research Institute of Petroleum and Petrochemicals, SINOPEC, Dalian 116045, China

**Keywords:** tin disulfide, carbon materials, composite nanomaterials, photocatalysis, energy storage batteries

## Abstract

Dimensional nanomaterials can offer enhanced application properties benefiting from their sizes and morphological orientations. Tin disulfide (SnS_2_) and carbon are typical sources of dimensional nanomaterials. SnS_2_ is a semiconductor with visible light adsorption properties and has shown high energy density and long cycle life in energy storage processes. The integration of SnS_2_ and carbon materials has shown enhanced visible light absorption and electron transmission efficiency. This helps to alleviate the volume expansion of SnS_2_ which is a limitation during energy storage processes and provides a favorable bandgap in photocatalytic degradation. Several innovative approaches have been geared toward controlling the size, shape, and hybridization of SnS_2_/Carbon composite nanostructures. However, dimensional nanomaterials of SnS_2_ and SnS_2_/Carbon have rarely been discussed. This review summarizes the synthesis methods of zero-, one-, two-, and three-dimensional SnS_2_ and SnS_2_/Carbon composite nanomaterials through wet and solid-state synthesis strategies. Moreover, the unique properties that promote their advances in photocatalysis and energy conversion and storage are discussed. Finally, some remarks and perspectives on the challenges and opportunities for exploring advanced SnS_2_/Carbon nanomaterials are presented.

## 1. Introduction

The fast depletion of fossil fuel and its environmental implications have led to the development of technologies for green-energy production and storage as well as environmental remediation. These technologies are of great interest to the research community to minimize carbon footprints. Therefore, breakthroughs in nanotechnology research could enable the creation of unique materials at the molecular level, opening up a slew of green industrial possibilities.

Dimensional nanomaterials have become a trendy topic in recent years and have aroused tremendous research interest due to their unique physicochemical and structural properties [1,2,3]. These dimensional nanomaterials have integrated architectures that exhibit well-oriented dimensions of zero-, one-, two-, or three-dimensional (0D, 1D, 2D, 3D) architectures, such as quantum dots, nanofibers, nanorods, nanowires, nanosheets, nanoflowers, and nanospheres [4,5,6]. This development has allowed for diverse applications in catalysis, optoelectronics, and electronic devices [7,8].

SnS_2_ nanomaterials have made impactful strides in the synthesis of dimensional nanomaterials, due to their unique hexagonal nanostructures and the ability to have sulfur chains with variable lengths. In addition, SnS_2_ has a favorable energy bandgap, low cost, low toxicity, excellent stability, and abundant reserves in nature. However, its wide application in batteries is hindered by low intrinsic conductivity and poor cycling stability [9,10]. One of the most effective techniques used to tackle these problems is the synthesis of SnS_2_ in composite nanomaterials. Carbon materials are economically abundant and have presented numerous advantages because of their unique physio-chemical and electrochemical properties, such as a high specific surface area, outstanding electrical and mechanical properties, and narrowing bandgap effect [11,12,13,14]. This makes carbon materials a great candidate to be used as a hybrid material. At the nano level, carbon materials offer a diversity of morphologies and structures (e.g., quantum dots, nanotubes, nanowire, graphene, nanospheres, graphene oxide, etc.), each of which is unique to its respective application technology [15,16,17,18,19]. The characteristic properties of composite nanostructures are inherited from the individual precursor components, leading to dimensionally synthesized hybrid architectures that are fit for various applications [20,21,22,23,24,25]. Moreover, the synergistic features of these functional nanocomposites can be achieved through the manipulation of their dimensions during synthesis. Therefore, a combination of SnS_2_ and carbon materials can lead to integrated SnS_2_/Carbon nanomaterials with enhanced properties in photocatalysis, electrochemical conversion, and energy storage applications. 

Progress has been made in the synthesis and applications of SnS_2_ nanomaterials using wet and solid-phase synthesis methods [26,27,28]. In the last five years, a number of exciting reviews on SnS_2_ have been published, focused on preparation, microstructure characterization methods, and application [29,30]. However, a focus on dimensional SnS_2_ nanocomposite architectures has not yet been reported. With the growing number of publications on SnS_2_ composite nanomaterials, there is a need to present an updated review article on the state-of-the-art development of SnS_2_/Carbon composites at the nano dimensional level. This review thus aims to give an overview of the progress made in the synthesis, dimensional characterization, and applications of SnS_2_/Carbon nanomaterials. SnS_2_ and SnS_2_/Carbon nanomaterials have some similarities in synthesis methods and application fields. So, the synthesis of SnS_2_ nanomaterials was first presented in this review for an overview of the fabrication methods, followed by SnS_2_/Carbon composite nanomaterials. The various nanostructural architectures were dimensionally classified in terms of zero, one, two, and three dimensions (0D, 1D, 2D, and 3D). Furthermore, this review examines the advances in the development of SnS_2_/Carbon hybrid nanomaterials in photocatalysis as well as electrochemical energy conversion and storage applications.

This review adopted a scoping review approach because it offers qualitative and quantitative opportunities to identify the scope of a body of literature relating to a particular topic, identify and clarify concepts associated with the research topic, and understand the research methods associated with the research topic [31]. The review used articles sourced from the Web of Science database starting with the keywords “SnS_2_”, “Tin disulfide”, “carbon”, “photocatal*”, and “batter*”. These were further enhanced by an iterative process of searching for articles around the three main focal areas that underpin this research, namely (i) synthesis, (ii) dimensional characteristics, and (iii) applications of synthesized material. This in turn became part of the criteria for selecting articles to be reviewed. In addition, all articles used were peer-review articles to ensure that the findings that are included in this review were based on sound science. Each article was reviewed to provide inputs for the three focal areas of this research. This subsequently informed the themes or categories which formed the sub-focal areas for this review. In situations where new themes were emerging, but the search did not capture more publications, further search was conducted. For instance, to identify additional and specific concepts related to the synthesis method, keywords such as wet or solid-state synthesis were applied to capture additional publications. This was the same when choosing articles with different morphological dimensions. As a result, there is no specific count of articles for each search and inclusion, which is typical of a scoping review.

## 2. Synthesis Methods

### 2.1. Wet Chemical Synthesis of SnS_2_ and SnS_2_/Carbon Nanomaterials

Wet chemical syntheses of nanomaterials involve chemical reactions in the solution phase using precursors at suitable experimental conditions. The synthesis technique varies depending on the solvent medium used. The wet chemical synthesis approach is a bottom-up method; as such, it offers a high degree of controlling and fabricating nanomaterials. Hydrothermal synthesis, [32,33] solvothermal synthesis, [34,35] template synthesis, [36] self-assembly, [37] hot-injection [38], and interface-mediated synthesis [39] all fall under wet-chemical synthesis routes. Amongst these, hydrothermal and solvothermal approaches are easy and reproducible methods and have been widely adapted for the preparation of inorganic nanomaterials.

#### 2.1.1. Wet Chemical Synthesis of SnS_2_ Nanomaterials

Several synthesis strategies have been reported, and new ones are being discovered to fabricate and better understand nanostructures of SnS_2_ nanomaterials. Through wet-chemical synthesis, Chaki et al. achieved 0D semiconductor SnS_2_ nanoparticles synthesized at room temperature using Tin(IV) chloride pentahydrate (SnCl_4_·5H_2_O) and thioacetamide (C_2_H_5_NS) as precursors [40]. Hexagonal crystal structures of SnS_2_ nanoparticles were also synthesized in a similar fashion without the addition of any surfactants or needing further purification [41].

As a typical wet-chemical synthesis method, hydrothermal treatment has often been used in the synthesis of SnS_2_. V-doped binary SnS_2_ buffer layers and SnS_2_ nanoflakes were prepared hydrothermally [42,43,44]. The obtained porous structures were interconnected with each other, displaying a high surface area. In other studies, using the solvothermal route, SnS_2_ nanomaterials were fabricated with different types of solvents [45,46]. This method has been used to achieve sheet-like, flower-like, and ellipsoid-like SnS_2_ nanostructures as potential electrode material [47]. The influence of thiourea concentration, solvent system, and reaction time have been proposed as vital in the solvothermal synthesis method. Wang et al. also added that high-boiling-point and low-viscosity solvents are needed for the reaction and product separation [48]. As such, the system can provide suitable surface energy that could effectively stabilize their 2D structures and suppress nanomaterials from further aggregation. In addition, using surfactants is a typical way to adjust the surface energy; as such, Triton X-100 was used, which played a crucial role in controlling the morphology of hexagonal SnS_2_ nanoflakes [49].

**Figure 1:**
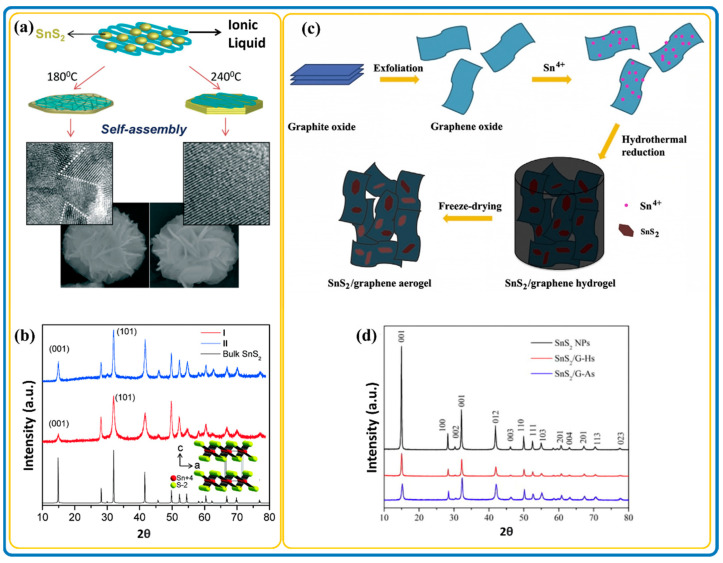
(**a**) Schematic illustration of ionothermal assembly (**b**) X-ray diffraction (XRD), of SnS_2_ flowers at lower and higher temperatures. Adapted with permission from [53]. Copyright 2014, Elsevier. (**c**) Self-assembly synthesis process (**d**) XRD, of 3D SnS_2_/Graphene aerogels. Adapted with permission from [54]. Copyright 2013, Elsevier.

Chemical vapor deposition (CVD) and the high-temperature hot injection method have also been successfully used to fabricate SnS_2_ nanostructures composed of vertically oriented 2D sheet arrays with high crystallinity and single-phased SnS_2_ nanosheets, respectively [50,51]. Solvents and precursors play important roles in catalyzing and increasing the kinetics of a reaction. Thus, for the controlled synthesis of nanomaterials, the focus should not only be on their fundamental shape or size-dependent properties and technological applications but also on the synthesis and assembly properties [52]. Another unique process was illustrated by Jana et al., using ionothermal synthesis to achieve SnS_2_ nanoflowers at low and high temperatures with exceptional nanostructures as depicted in Figure 1a [53]. The crystal structures of the synthesized nanostructures were determined by XRD analysis, highlighting the hexagonal SnS_2_ structures with (001) and (101) crystallographic planes (Figure 1b). This hexagonal nature is common in SnS_2_ and composite associations. The medium for synthesis was water-soluble ionic liquids. The ionic liquid served as a template at a low temperature to achieve the hierarchical layered polycrystalline 2D SnS_2_ nanosheet petals. These were combined by the effects of hydrogen bonding, imidazolium stacking, and electrostatic and hydrophobic interactions. On the other hand, a high-temperature reaction yielded plate-like nanosheets with well-defined crystallographic facets because of the rapid inter-particle diffusion across the ionic liquid. The various synthesis processes of SnS_2_ nanomaterials have allowed the integration of diverse hybrid materials to enhance their application properties. 

#### 2.1.2. Wet Chemical Synthesis of SnS_2_/Carbon Nanomaterials

Just like SnS_2_ nanomaterials, the structure of SnS_2_/carbon composite nanomaterials depends on the precursors and the synthesis conditions. Normally, SnS_2_/carbon composites result in different dimensional architectures, where the SnS_2_ nanostructures anchor themselves onto interpenetrated carbon materials with varied architectures. Figure 1c is a clear representation of self-assembled SnS_2_/carbon composite nanostructures. It shows 3D SnS_2_/graphene aerogel nanostructures fabricated through in situ macroscopy self-assembly using a hydrothermal process, followed by freeze-drying to preserve its 3D architectures. Figure 1d shows the crystalline structure of SnS_2_ in composite materials; however, it could not detect the carbon peaks due to its amorphous form. For that matter, Raman spectroscopy was proposed for the detection of carbon in the composite. 

Controlling the growth orientations of SnS_2_ nanostructures on nanocarbon surfaces has been reported as a challenging concept as seen in the fabrication of parallel and vertically aligned SnS_2_ nanostructures on graphene nanosheets [55]. Hence, an understanding of the mechanism of SnS_2_/carbon hybrid synthesis, with desired properties and varied nanostructures, is important in nanotechnological applications. Liu et al. synthesized SnS_2_/bacterial-cellulose-derived carbon nanofiber (BC-CNF) first by the hydrolysis of thioacetamide, followed by in situ metathesis reactions, and finally by self-assembly and oriented crystallization processes [56]. The resultant BC-CNFs had a highly porous 3D network with an average diameter of 30–50 nm. Among the methods adopted for synthesizing SnS_2_/Carbon nanomaterials, the most prevalent is the hydrothermal process. This technique can enhance the characteristics and stability of nanomaterials while concurrently controlling the structures of the hybrid composites. These allow for interconnected networks with a high surface area which enhances the synergetic interactions between the layered SnS_2_ and the carbon by increasing their contact areas [57]. Furthermore, the interconnected network helps SnS_2_ in alleviating the mechanical stress, preventing its aggregation, and accommodating its volume change during cycling [58,59]. For instance, SnS_2_/graphene oxide nanocomposites were synthesized by reflux condensation together with a hydrothermal strategy using an anionic surfactant, sodium dodecyl sulfate (SDS) [60]. Zhang et al. also proposed a means for fabricating hierarchical polyaniline/SnS_2_@carbon nanotubes onto the carbon fiber (CF) surface [61]. However, synthesis limitations, such as low pressure and temperature can seriously affect the rate performance of composite materials [62,63].

Solvothermal synthesis has been used to investigate the synthesis of SnS_2_/carbon composite nanomaterials, but only a handful of reports exist on the use of this method. For example, Zhang et al. synthesized one-pot flexible SnS_2_/CNT (2D nanosheet/3D self-assembled flower) composites which were controlled by a time-dependent process [64]. Moreover, functionalized graphene sheets (FGS) were used to synthesize graphene-SnS_2_ nanocomposites via a solvothermal method [65]. In most cases, annealing is used for further treatment to improve the phase purity and crystallinity of nanomaterials before use in various applications [10,66].

### 2.2. Solid-Phase Synthesis of SnS_2_ and SnS_2_/Carbon Nanomaterials

Solid-phase synthesis is a top-down approach to synthesizing inorganic nanomaterials. The procedure involves milling and may include many annealing steps with several intermediate milling procedures to heighten the uniformity of the mixture and reduce the sizes of the fabricated materials [67,68,69,70,71]. Additional milling tends to make the particles more sinter active in the heat treatment procedures that follow [72,73]. Furthermore, huge quantities of materials can be synthesized in a reasonably straightforward method, but the resulting nanomaterials have a comparatively high agglomeration compared to the wet synthesis processes discussed above [74,75]. As a result, solid-phase synthesis produces relatively large particle sizes and poor homogeneity which are somewhat unavoidable.

#### 2.2.1. Solid-Phase Synthesis of SnS_2_ Nanomaterials

Solid-phase synthesis of SnS_2_ nanomaterials is an alternative fabrication method that helps the growth of SnS_2_ nanostructures by supplying an adequate amount of precursors [76]. Usually, this is carried out without the aid of a template, inert gas protection, or a vacuum environment but by heating the solid precursor mixtures in air at certain temperatures and time followed by washing treatment [77]. In some reports, SnS_2_ nanoflakes were fabricated using a suitable amount of SnCl_4_·5H_2_O and thiourea mixed and grounded thoroughly until a homogeneous mixture was acquired and subsequently heated in a crucible [78,79]. Owing to the intrinsic anisotropic nature of SnS_2_ crystals, solid-phase synthesis tries to enhance its surface area to achieve desired nanostructures through the milling process. Xiao et al. and Wang et al. prepared SnS_2_ nanomaterials by heating the precursors at their liquid–solid phase, i.e., at the melting points and boiling points of tin (Sn), sulfur (S), and ammonium chloride (NH_4_Cl) in air [80,81]. In addition, the presence of NH_4_Cl aided and promoted the synthesis of pure SnS_2_ under mild conditions. It is worth noting that the annealing process of nanomaterials can also bring about self-purification due to the impurities and intrinsic material defects that prefer moving toward the surface during the annealing process. 

#### 2.2.2. Solid-Phase Synthesis of SnS_2_/Carbon Nanomaterials 

**Figure 2:**
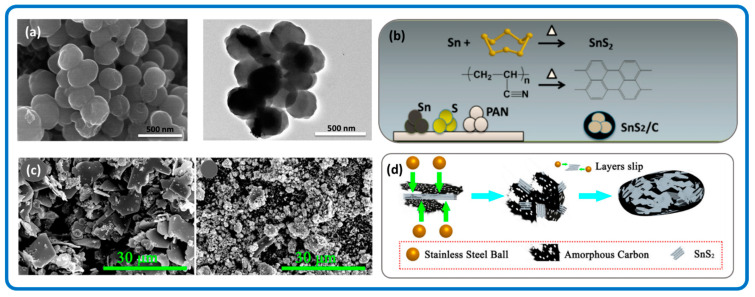
(**a**) SEM and TEM images, (**b**) Schematic illustration of solid-state synthesis route, of SnS_2_/carbon nanomaterials. Adapted with permission from [82]. Copyright 2015, American Chemical Society. (**c**) SEM images of un-milled and directly milled SnS_2_/Carbon. (**d**) Schematic illustration of ball milling of SnS_2_/carbon to decrease its crystallinity. Reprinted with permission from Springer Nature from [83]. Copyright 2016, Elsevier.

To the best of our knowledge, limited literature exists on the solid-state synthesis of SnS_2_/Carbon nanomaterials. The solid synthesis of SnS_2_/Carbon nanomaterials may involve microwave heating, calcination, milling, or a combination of these processes to achieve a homogeneous crystalline product. The mechanical energy used creates phase transformations and chemical reactions at very low temperatures. For instance, ball milling enables the reduction in particle sizes and characteristic lengths in addition to the effective mutual dispersion of the processed phases. Wang et al. synthesized a SnS_2_/Carbon composite by annealing metallic Sn, S powder, and polyacrylonitrile (PAN) mixed in a sealed glass tube under vacuum at 600 °C for 3 h [82]. This resulted in SnS_2_ nanostructures embedded in the carbon matrix that was generated by the carbonization of PAN. The morphologies are shown in Figure 2a and the synthesis process is schematically shown in Figure 2b. Furthermore, the synthesized SnS_2_/carbon composite was directly milled in NaCl which reduced the crystal structure of the SnS_2_/Carbon nanocomposite, and this improved the overall battery performance of the synthesized SnS_2_/Carbon nanomaterial [83]. Figure 2c shows the SEM of un-milled and directly milled SnS_2_/Carbon structures, respectively and Figure 2d shows the schematic fomation of SnS_2_/Carbon composite.

**Figure 3:**
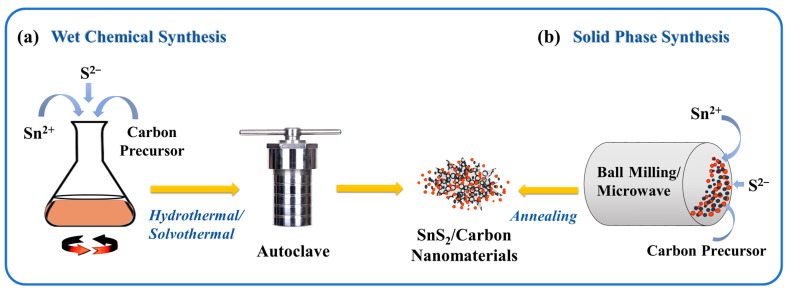
(**a**)Schematic illustration of wet chemical synthesis and (**b**) solid-state synthesis route of SnS_2_ and SnS_2_/Carbon nanomaterials.

In some other processes, solid-state synthesis has been indicated to require low temperatures and help to improve the purity of the resultant substances [84,85]. The solid-state syntheses have also been applied in the synthesis of other SnS_2_/composites including Phosphorus-SnS_2_ composites, which is not the focus of this review [86]. Figure 3a demonstrates the simple synthesis routes of SnS_2_/Carbon nanomaterials by wet chemical and Figure 3b by solid-state synthesis. Table 1 shows the various SnS_2_/Carbon nanomaterials achieved through wet and solid-state synthesis approaches. In other instances, a hybrid synthesis method was employed to achieve SnS_2_/carbon composite nanomaterials [87,88]. In one instance, the carbon precursor was synthesized at elevated temperatures before it was further combined with the Sn^2+^ and S^2−^ precursors to form the composite SnS_2_/carbon nanomaterials [89,90].

## 3. Dimensional Characteristics of SnS_2_ and SnS_2_/Carbon Nanomaterials

Nanomaterials possess a variety of shapes and sizes. In some cases, their names are generated and characterized by their shapes or orientations. For example, nanospheres are spherical, nanotubes are tube-shaped, etc. Nanostructure classifications are also based on their dimensions, compositions, uniformity, and agglomeration. Classification based on dimensionality is a generalization of the concept based on the aspect ratio of 0D, 1D, 2D, or 3D. These dimensions or morphologies result from a variety of precursors, temperature, pH, templates, the mode of reagent dosage during synthesis, etc. The ability to control the morphology of nanomaterials is crucial in exploiting their properties for applications. As a measure of the dimensional characteristics of SnS_2_ and SnS_2_/Carbon nanomaterials in this review article, TEM and SEM analyses were mainly used.

### 3.1. Dimensional Characteristics of SnS_2_ Nanomaterials

#### 3.1.1. Zero-Dimensional (0D Nanodots) SnS_2_ Nanomaterials

**Figure 4:**
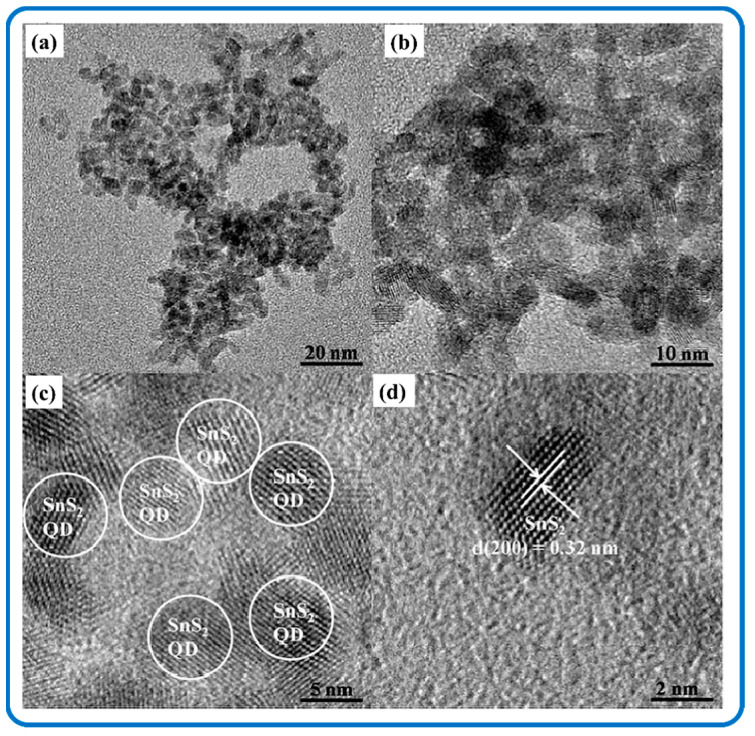
TEM images of SnS_2_ QDs (**a**,**b**); HR-TEM images of SnS_2_ QDs (**c**,**d**). Reproduced with permission from [118]. Copyright 2016, Elsevier.

SnS_2_ quantum dots (QDs) possess strong luminescence, good aqueous stability, and biocompatibility. Therefore they are often used in the field of sensing and biology [115]. Excitation and emission properties exhibited by SnS_2_ QDs were credited to the polydispersity of SnS_2_-QDs and its characteristic feature of quantum confinement and edge effects [116]. Negatively charged SnS_2_ QDs were made by inserting electrons into vacant molecular orbitals, whereas positively charged SnS_2_ QDs were made by injecting holes into the highest occupied molecular orbitals, and these collided with the stable SnS_2_ QDs to produce excited SnS_2_ QDs that could emit light [117]. Figure 4a–d show representative transmission electron microscopy (TEM) and high-resolution transmission electron microscopy (HRTEM) details of SnS_2_ QDs. Figure 4a,b are nearly monodispersed SnS_2_ QDs with a mean size of 6.5 nm. A single particle lattice spacing of 0.32 nm is seen in Figure 4d corresponding to the (200) plane of hexagonal SnS_2_ [118].

The surface energy and crystal structures of the SnS_2_ QDs are dependent on synthesis conditions. Optimized synthesis conditions could result in a significant increase in the surface-to-volume ratio and influence the surface energy and phase stability greatly. Hydrothermally synthesized SnS_2_ QDs are in situ functionalized and pH sensitive [115,119]. In application, these nanomaterials can connect and partly fuse to adjacent ones leading to much flatter structures after annealing. In the end, this is valuable to form good contact between the active layer and the electrode material [120].

#### 3.1.2. One-Dimensional (1D) SnS_2_ Nanomaterials

**Figure 5:**
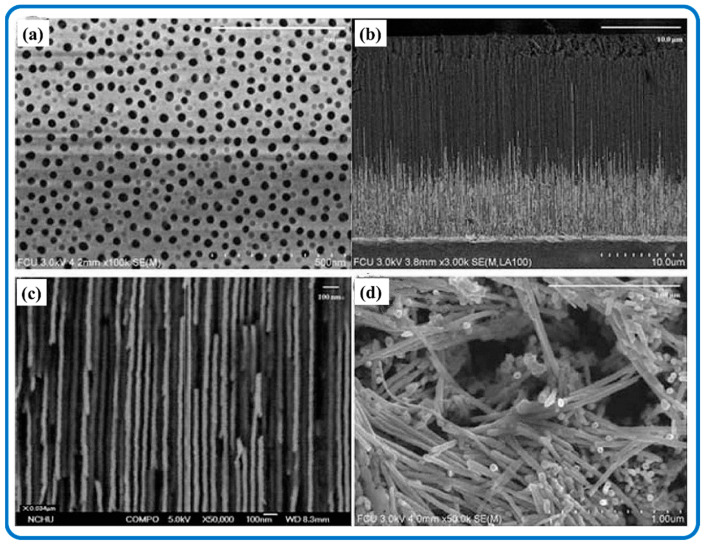
Field Emission Scanning Electron Microscope (FE-SEM) micrographs of (**a**) top view of the AAO templates for SnS_2_ nanowire formation, (**b**) cross-section view of SnS_2_ nanowires embedded in an AAO template, (**c**) the magnified FE-SEM micrograph of SnS_2_ nanowires, and (**d**) SnS_2_ nanowires detached from the AAO templates. Reproduced from [121]. 2009, Springer. CC BY 2.0.

One-dimensional nanostructures are of interest due to their potential to serve as the basis for determining the size and dimensionality dependence of a material’s physical properties. Many solid structures of chalcogenide grow from 1D nanostructures. One-dimensional nanomaterials have been exploited as a novel model while investigating the size and dimensional dependence of functional properties. They also play an important role as interconnected nanostructures and as the key units in fabricating electronic, optoelectronic, and electrochemical energy devices with nanoscale dimensions. SnS_2_ nanowires were synthesized by sulfurizing the Sn nanowires, which were embedded in the nanochannels of anodic aluminum oxide (AAO) templates. The characterization of these nanowires is shown in Figure 5 [121]. After detaching from the AAO templates, SnS_2_ nanowires achieved a diameter of about 40 nm. It is worth noting that reports on the synthesis of 1D nanomaterial are rare, owing to the fact that most synthesized SnS_2_ nanomaterials are the building blocks for achieving other dimensionally structured nanomaterials.

#### 3.1.3. Two-Dimensional (2D) SnS_2_/Flake Nanomaterials 

**Figure 6:**
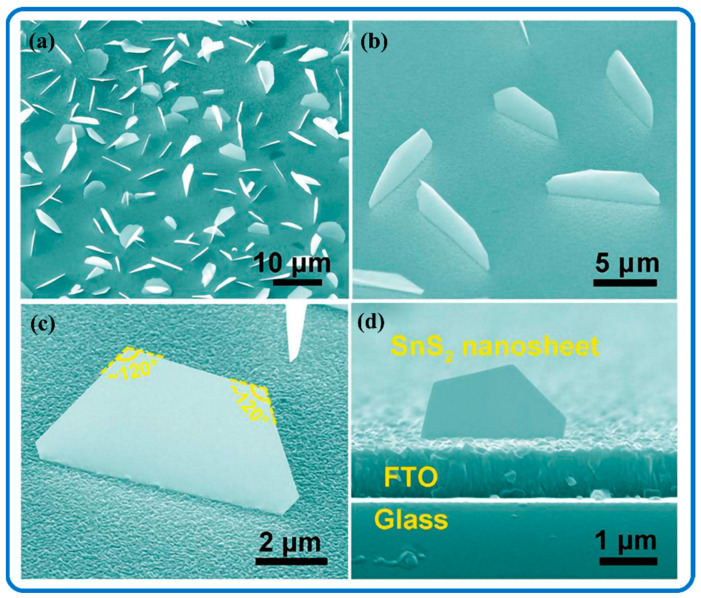
(**a**,**b**) Low-magnification SEM images. (**c**) High-magnification SEM image of vertical SnS_2_ nanosheet taken at 45° from a normal viewing angle. (**d**) Typical cross-sectional-view SEM image of one vertical SnS_2_ nanosheet grown on FTO substrate. Reproduced with permission from [126]. Copyright 2017, Royal Society of Chemistry.

Researchers have made significant advances in the preparation, characterization, adjustment, and theoretical investigation of 2D materials. The abundance of 2D materials has elevated them with a range of material frameworks in methodological studies for the development of nano- and atomic-level applications. Two-dimensional SnS_2_ nanocrystals exhibit semiconductor characteristics, [122] owing to their high carrier movement and large bandgap [123]. SnS_2_ has a sandwich-like structure with an S plane held in between two Sn planes, all in hexagonal order. The adjacent sulfur atoms in the sulfur layers are bonded, allowing for easy layer separation via chemical or mechanical exfoliation [41]. However, vacancy defects in 2D SnS_2_ nanomaterials are known to have a major influence on material characteristics and are unavoidable during exfoliation [124]. Sun et al. confirmed the formation of micrometer-sized SnS_2_ nanosheets with exposed (011) facets as the primary surfaces [125]. These 2D nanosheets could be reconstructed by lateral confinement with longitudinal extension, and a typical 2D SnS_2_ structure is displayed in Figure 6. The as-grown SnS_2_ nanosheets were quasi-vertically oriented and standing free on the fluorine-doped tin oxide (FTO) substrate (Figure 6a–c). The SnS_2_ nanostructures displayed a well-defined semi-hexagonal shape. Similarly, Li et al. achieved 2D SnS_2_ nanoflakes grown perpendicular to the substrate in a low-temperature zone of a SiO_2_/Si substrate [123]. Strong light absorption, short minority-carrier transport distances, and a wide exposed surface area for catalytic reactions have all helped 2D SnS_2_ nanomaterials to effectively harvest photocurrent [50]. Furthermore, 2D SnS_2_ with characteristics such as mono-dispersity, high compactness, open morphology, well-defined structures, and maximally exposed surfaces/edges are favorable [51].

#### 3.1.4. Three-Dimensional (3D) Self-Supporting SnS_2_ Nanoflowers 

**Figure 7:**
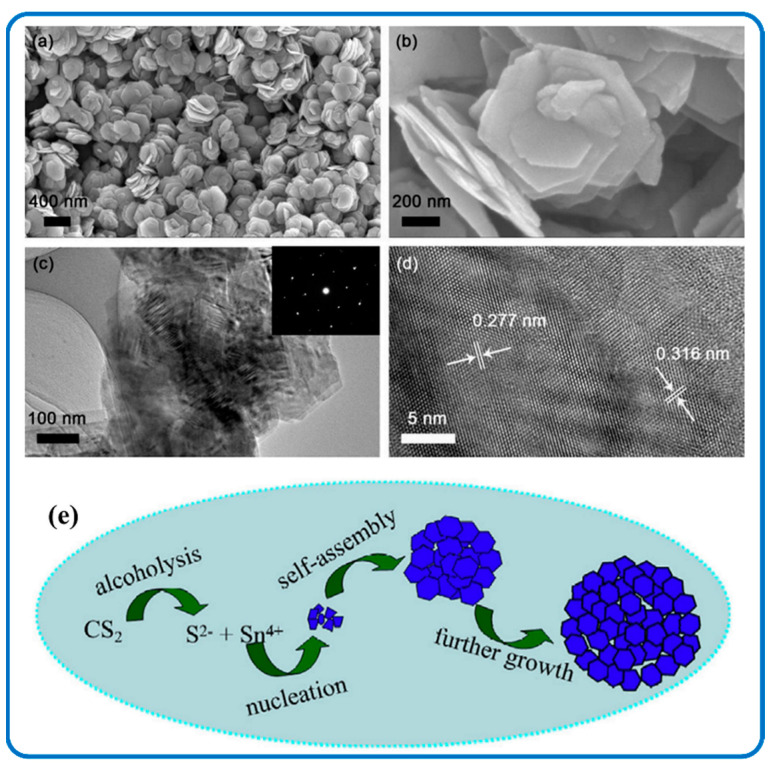
(**a**,**b**) SEM, (**c**) TEM images, and (**d**) Fringe interval of the as-prepared SnS_2_ nanoflowers. Reproduced with permission from [128]. Copyright 2018, Elsevier. (**e**) Schematic illustration of 3D SnS_2_ nanoflowers. Reproduced with permission from [129]. Copyright 2013, Elsevier.

Three-dimensional SnS_2_ nanomaterials have hierarchical flower-like architectures with nanosized building blocks and a complex assembled architecture. Their large surface area can reduce the concentrated polarization and offer more sites for accommodation [127]. Figure 7a,b are flower-shaped nanostructures analyzed by TEM and SEM analysis. Figure 7c,d represents the TEM images of SnS_2_ nanoflowers and the fringe interval with the d-spacing of hexagonal SnS_2_, respectively. The schematic approach leading to the formation of SnS_2_ nanoflowers is demonstrated in Figure 7e [128,129]. Xiong et al. also described well-defined SnS_2_ nanoflowers for NH_3_ detection by a facile solvothermal method [130]. On the other hand, 3D hierarchical SnS_2_ microspheres consisting of thin-layered nanosheets were synthesized via a one-pot hydrothermal method [131]. By altering the ratio of SnCl_4_ to L-cysteine, they were able to keep their morphologies under control. In addition, mild hydrothermal treatment in the presence of octyl-phenol-ethoxylate (Triton X-100) at 160 °C led to the achievements of 3D nanoflowers with a spot-like appearance along the [010] axis of the SnS_2_ crystal [132].

### 3.2. Dimensional Characteristics of SnS_2_/Carbon Composite Nanomaterials

The hybrid structures of SnS_2_/Carbon materials at the nano level have permitted the control of desired properties and features. Table 2 suggests the possible hierarchical formation of SnS_2_/Carbon composite nanomaterials. It tries to depict the synergies between SnS_2_ nanostructures and carbon nanomaterials in the hybridized structures. The concept of composites further tries to enhance the nanomaterials’ internal and external capabilities as well as the physical/chemical compatibility. The unique properties of these composite nanomaterials are of great interest for their environmental and energy storage applications.

#### 3.2.1. Zero-Dimensional (0D) SnS_2_/Carbon Composite Nanodots 

Various questions come up about what constitutes 0D hybrid materials. The literature captures this concept of zero-dimensional composites giving preference to one component. The individual nanostructures of SnS_2_ and carbon have achieved great heights in many applications, for example, in the fields of photoelectric detectors, solar photocatalysts, and photovoltaic solar cell applications. To the best of our knowledge, there are no reports on zero-dimensional composite SnS_2_/Carbon nanomaterials. SnS_2_/Carbon nanostructures could result from a combination of SnS_2_ QDs and Carbon QDs in a hybridized synthesis approach. The achievement of this structural material at a low dimension has proven beneficial in its application in catalysis and electrochemistry [133]. Chen et al. found that the tiny size of SnS_2_ QDs makes them easy to insert into graphene nanosheets which prevents the restacking of graphene nanosheets [134]. Meanwhile, the inserted SnS_2_ QDs showed an enhanced photocatalytic effect. The carbon dots-SnS_2_ nanomaterials could show excellent photocatalytic adsorption capacity by acting as a good electron acceptor. Therefore, a synergetic combination of SnS_2_ QDs and carbon QDs could be a powerhouse for future applications.

**Table 2:** Schematic comparison of different hybrid nanostructures of SnS_2_/Carbon composite nanomaterials.

Dimension	SnS_2_ Structures	Carbon Structures	Published Composite Nanomaterials	Ref.
0D	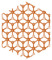	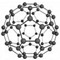	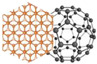	[91]
1D	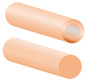	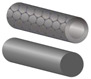	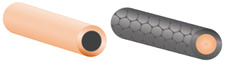	[58,92,95,135,136,137,138]
2D	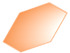	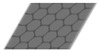	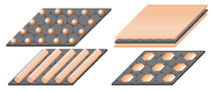	[89,139,140,141,142,143]
3D	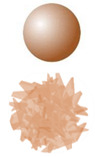	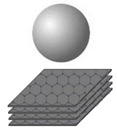	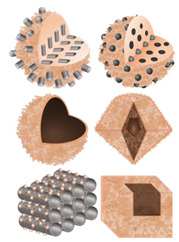	[111,136,144,145,146,147,148,149,150]
Potential structures of SnS_2_/Carbon composite nanomaterials				
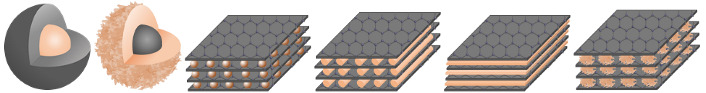				

The schematic images in Table 2 were adapted withpermissions from [58], copyright 2011, American Chemical Society; [92], copyright 2018, Elsevier; [135], copyright 2014, Royal Society of Chemistry; [136] copyright 2018, Elsevier; [138], copyright 2016, Royal Society of Chemistry; [89], copyright 2017, Elsevier; [139], copyright 2019, Zhang et al. (CC BY); [140], copyright 2019, American Chemical Society; [141], copyright 2020, American Chemical Society; [142], copyright, 2017, American Chemical Society; [143], copyright 2011, Royal Society of Chemistry; [111], copyright 2017, Elsevier; [144], copyright, 2020 Wiley-VCH; [145], copyright 2021, Elsevier; [146], copyright 2021, Elsevier; [148], copyright 2015, Elsevier; [149], copyright 2019, (CC BY 4.0), [150], copyright 2021, (CC BY-NC-ND 4.0).

#### 3.2.2. One-Dimensional (1D) SnS_2_/Carbon Composite Nanomaterials

**Figure 8:**
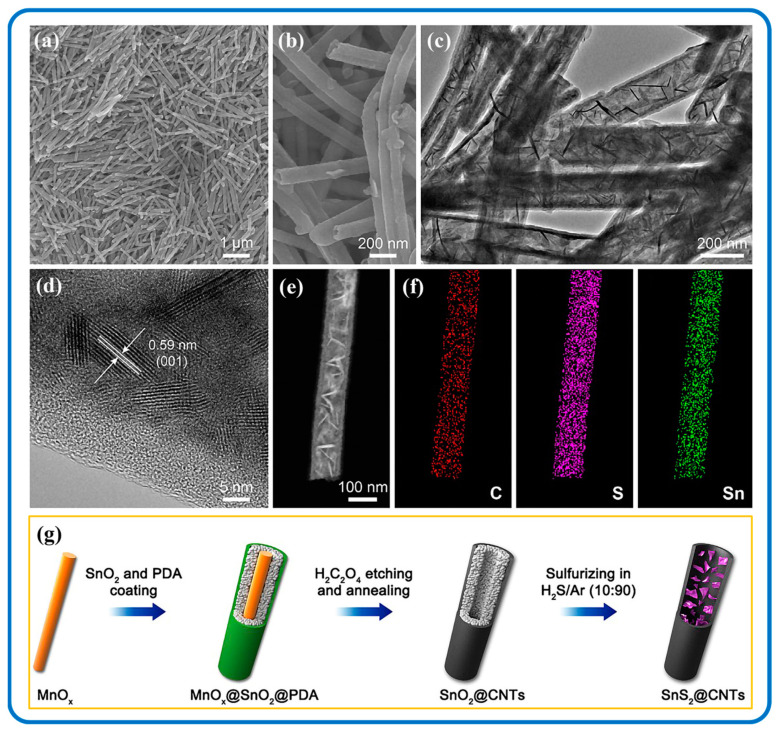
(Characterization of SnS_2_@CNTs in terms of morphology and structure, (**a**,**b**) FE-SEM images, (**c**) TEM image, (**d**) HRTEM image showing SnS_2_ nanosheet with evident lattice fringe space of 0.59 nm, (**e**) High-angle annular dark-field scanning transmission electron microscope (HAADF-STEM) image of one individual SnS_2_@C nanotube, and (**f**) elemental mapping of SnS_2_@C nanotube, corresponding to C, S, and Sn elements. (**g**) Synthesis of SnS_2_@CNTs depicted schematically. Reproduced with permission from [136]. Copyright 2018, Elsevier.

Controlling the orientation and polymer chain alignment of 1D nanostructures can increase their multifunctional features such as thermal and electrical conductivity [151]. The characteristic properties of hierarchical 1D composite nanomaterials are usually realized by using one of the components as a backbone or template material, and the other component is deposited on the surface or within it [152,153,154]. An understanding of 1D nanostructures has been intensively covered by Wei et al. [155], where the fabrications and applications of 1D mono and hybrid nanomaterials are touched on. In most 1D SnS_2_/Carbon hybrid formations, the SnS_2_ part, usually nanosheets or nanoflakes is embedded in the 1D carbon template parallelly or inclined at an angle. This enhances the properties of SnS_2_/Carbon nanomaterials and correspondingly influences their applications [138]. SnS_2_/CNT composite nanomaterials are gaining attention because of their large surface area and the improved conductivity compared to SnS_2_. The sheet size of SnS_2_ is greatly reduced when it is clustered in SnS_2_/CNT hybrid nanocomposites, indicating that the introduction of CNTs refined the sheet size of SnS_2_ [156]. This leads to the CNTs evenly wrapped on the surface of or interspersed in the SnS_2_ sheets which is beneficial for improving the conductivity of the SnS_2_. In addition, SnS_2_/CNTs can be attached to the surface of the separators without any peeling and blanking, thus showing good flexibility and mechanical stability [94,157]. In an extraordinary case, the CNTs could act as templates for SnS_2_ materials. Jin et al. demonstrated this by filling hard CNT templates with Sn materials and sulfurizing beyond 300 °C to achieve SnS_2_ nanostructures as a dominant phase within the CNTs [97]. Again, the diffraction peak corresponding to the (001) plane of SnS_2_/CNT hybrid nanostructures exhibits preferentially oriented growth along this plane. Figure 8 demonstrates a typical formation of 1D SnS_2_/Carbon composite nanostructure through a facile templating synthesis using MnO_x_ nanorods as templates [136]. By adjusting the sulfurization temperature, it aided in the structural control during the formation of the nanocomposite, such that the SnS_2_ nanosheets were encapsulated in amorphous carbon nanotubes. 

**Figure 9:**
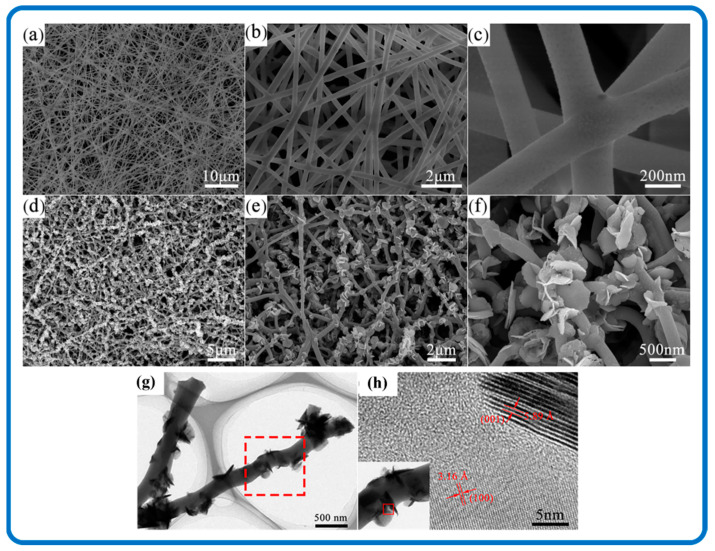
(**a**–**c**) Different-magnification FE-SEM images of carbon nanofibers and (**d**–**f**) SnS_2_/NSDC nanofibers. (**g**) TEM image of SnS_2_/NSDC nanofibers. (**h**) HRTEM image of SnS_2_/NSDC nanofibers marked in the red area. Reproduced with permission from [92]. Copyright 2018, Elsevier.

The carbon nanofiber network has also made feasible contributions in terms of SnS_2_/Carbon nanomaterial formation. For instance, Xia et al. prepared SnS_2_ embedded in nitrogen and sulfur dual-doped carbon (SnS_2_/NSDC) nanofibers by a facile electrospinning technique as indicated in Figure 9 [92]. Figure 9d–f illustrates the various morphological features of the SnS_2_/Carbon composite nanofibers. The carbon nanofiber framework provides a conductive host and is tolerant to the volume variation of SnS_2_ during the charging/discharging processes, thereby maintaining the structural stability of the SnS_2_/Carbon electrode [158]. Furthermore, the microstructures of SnS_2_ nanosheets can provide rich migration paths of sodium ions and electrons; therefore, the hybridized synergy realizes a rapid and efficient electron transport, which leads to an enhanced performance of the SnS_2_/Carbon system [159,160,161,162].

#### 3.2.3. Two-Dimensional (2D) SnS_2_/Carbon Composite Nanomaterials

The sheet-like nature of 2D nanomaterials makes them attractive for resolving diverse application demands. Coupled with SnS_2_, 2D SnS_2_/carbon nanomaterials display a wide range of extraordinary properties especially in alleviating volume expansion [163]. Especially, minimal stacking of 2D layered materials can be achieved for better application performance due to the introduction of the conductive graphene layers, which conveniently protects the SnS_2_ nanosheets from breakdown and weakens their agglomerating and re-stacking trends. Through an all-solid-state synthesis approach, Lonkar et al. achieved the minimal stacking of SnS_2_ nanosheets and realized a scalable 2D SnS_2_ and graphene layered nanosheets (SnS_2_/G) via ball milling using robust mixed precursors and sufficient metal-sulfur intercalation within the GO substrate [141]. Furthermore, it showed great inherent conductivity, high specific surface area, and high catalytically active planes, which is a plus in battery applications.

**Figure 10:**
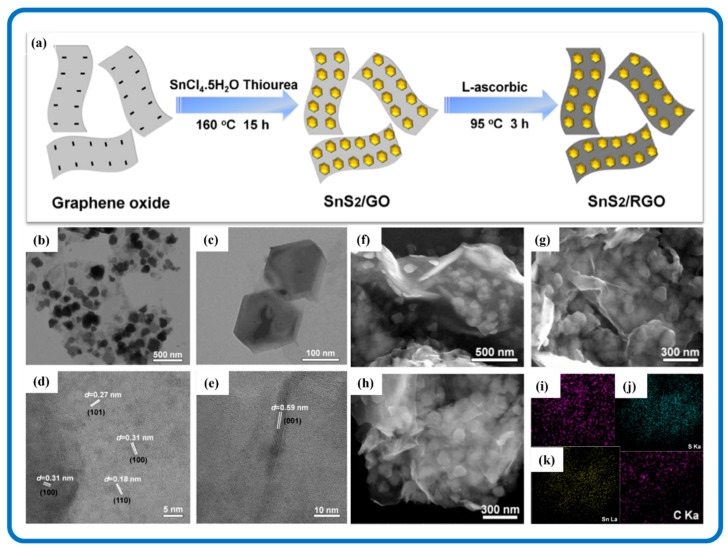
(**a**) Schematic illustration of the fabrication process for the SnS_2_/rGO composite, (**b**,**c**) TEM and (**d**,**e**) HRTEM images of the SnS_2_/rGO composite, (**f**–**h**) SEM images of the SnS_2_/rGO composite, and (**i**–**k**) the elemental mapping of C, S, and Sn, respectively, corresponding to (**h**). Adapted with permission from [165]. Copyright 2017, Elsevier.

Two-dimensional nanostructures are considered as architectural building blocks to hasten reaction kinetics and shorten the transport paths of electrons and ions. Therefore, the 2D synergetic combination of SnS_2_ with 2D carbon materials would be vital in enhancing its application. For example, SnS_2_ itself experiences low catalytic and electrical activity, but the existence of strong interfaces between SnS_2_ and graphene might facilitate and ease charge transportation [164]. Furthermore, the carbon component serves as a bridge for SnS_2_ nanomaterials which serves as a transfer highway to improve the efficiency of charge transportation. This has proven to be beneficial in improving the overall charge transportation of the resulting nanocomposites. Figure 10 shows fabricated 2D SnS_2_ nanoplates anchored on rGO nanosheets by a one-step controllable hydrothermal synthesis approach followed by a slight reduction reaction [165]. The face-to-face (FTF) nanostructure allowed for a large contact area, which improved the composite’s conductivity and reduced the migration distance of Na^+^ and electrons between rGO and SnS_2_. 

**Figure 11:**
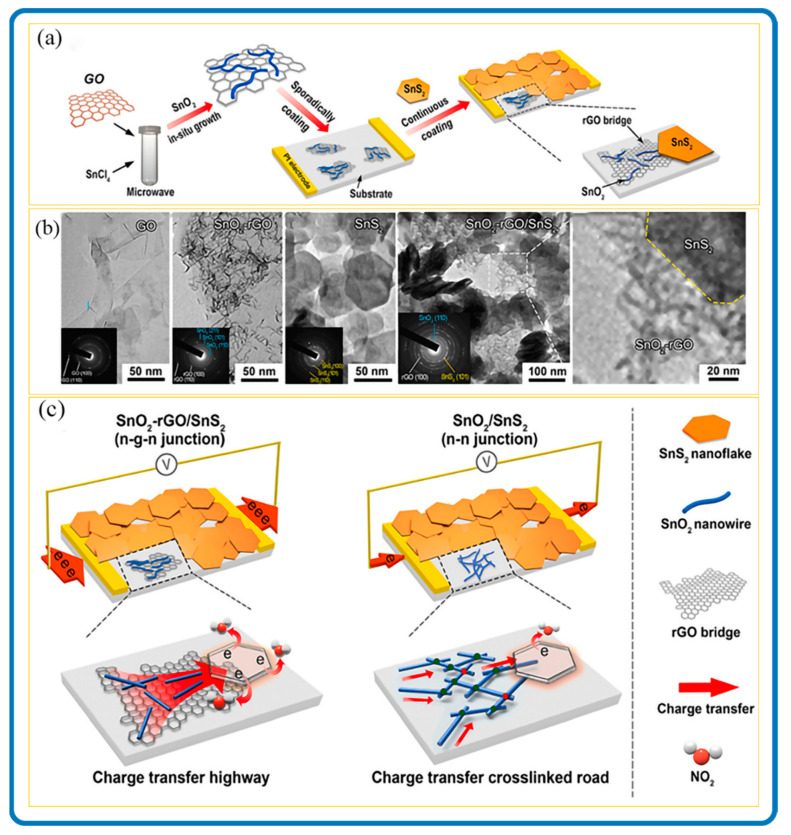
(**a**) Schematic diagram of the construction of ternary SnO_2_-rGO/SnS_2_ gas sensor with n-g-n junctions. (**b**) Subsequent TEM images. (**c**) Schematic images of charge transfer modification between SnO_2_-rGO/SnS_2_ sensor with novel n-g-n heterojunctions and SnO_2_/SnS_2_ sensor with traditional n-n junctions. Reproduced with permission from [104]. Copyright 2021, Elsevier.

In addition, the charge transfer resistance tests of 2D nanocomposites demonstrated superior transportation kinetics as shown in Figure 11, which may originate from the fast electron transport of FTF SnS_2_/Carbon composites [104]. These features have also been seen in photocatalytic SnS_2_/Graphene hybrid nanosheets with identically 2D structural configurations where SnS_2_ nanoplates were evenly distributed across the graphene framework [166]. Wang et al. used mixed processes of hydrothermal and vapor-phase polymerization to successfully produce triaxial nanocables of conducting polypyrrole@SnS_2_@carbon nanofiber (PPy@SnS_2_@CNF) [93]. The nanostructures showed a porous and interconnected nanofiber network with outstanding battery application.

#### 3.2.4. Three-Dimensional (3D) Self-Supporting SnS_2_/Carbon Nanomaterials

**Figure 12:**
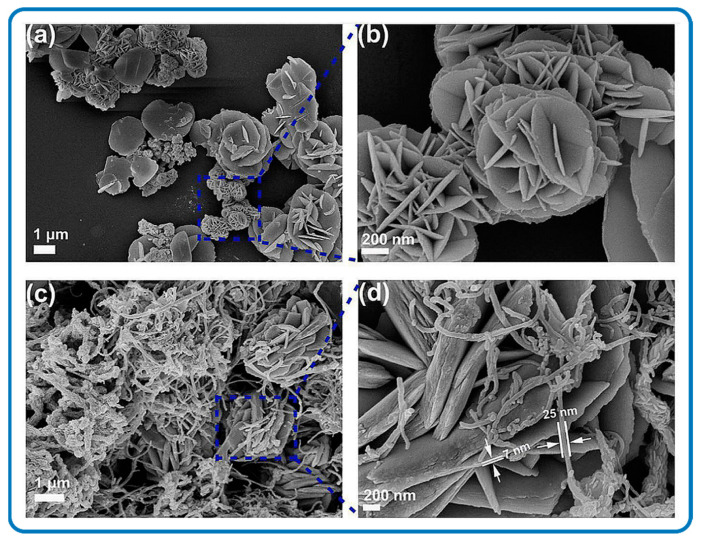
SEM images of (**a**,**b**) SnS_2_ and (**c**,**d**) SnS_2_/CNTs. Reproduced with permission from [111]. Copyright 2016, Elsevier.

Three-dimensional nanostructures of SnS_2_/Carbon nanomaterials are usually not confined to the nanoscale in any dimension. Three-dimensional nanostructures offer appreciable expanded levels of functionality compared to 2D counterparts because the strains of the 3D shape can induce bending and twisting below the maximum endurance limit for each layer in the construct [167]. Three-dimensional SnS_2_/Carbon composite nanostructures could result from different synthesis approaches with different combinations of SnS_2_ and carbon precursors. In general, 0D, 1D, and 2D nanomaterials are the building blocks to achieving desired structural nanocomposites. The dispersions of the nanomaterials could include, for example, nanodots, nanotubes, or nanosheets as well as multi-nano layers. These structural elements are usually in close contact with each other, thereby resulting in 3D interfaces. Many 3D nanocomposite combinations have been reported in the literature [168,169,170]. For example, through the hydrothermal synthesis method, carbon nanotubes formed a cross-winding network on the surface of SnS_2_ nanoplates. This resulted in flower-like SnS_2_/Carbon composite nanostructures via electrostatic interactions as shown in Figure 12a–d [111]. The diameter of the CNTs was 25 nm with a length of 1–3 µm (Figure 12d). The hybridized 3D SnS_2_/Carbon structures could alleviate the internal stress induced by the volumetric expansion/contraction during Li^+^ insertion/extraction processes [148]. Liu et al. obtained uniform 3D interpenetrating porous membrane nanostructures of SnS_2_/Carbon fabricated via non-solvent-induced phase separation (NIPS) membrane technology, and this technology offered an abundant membrane pore space for uniform SnS_2_ nanosheet development via C–S covalent bonding [171].

**Figure 13:**
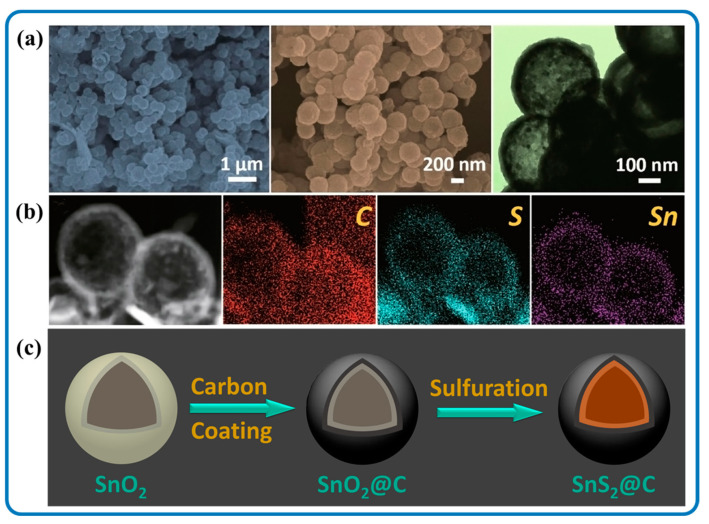
(**a**) TEM images of composite SnS_2_/C, (**b**) elemental mapping images of C, S, and Sn, (**c**) Schematic illustration of SnS_2_/carbon nanocomposite fabricated from SnO_2_. Reproduced from [112]. Copyright 2019, Springer, Open Access.

Aside from 3D network composite nanomaterials, hollow 3D composite nanostructures are quite common and have shown unique properties in energy storage fields. Li et al. reported hollow 3D SnS_2_/Carbon nanospheres that were designed through a facile solvothermal route followed by an annealing treatment (Figure 13a). The SnS_2_/Carbon nanocomposite resulted from using SnO_2_@C hollow nanospheres as a template and thioacetamide as a sulfur source as shown in Figure 13c. Moreover, the hollow structure and morphology were maintained during the synthesis process. The 3D SnS_2_/Carbon nanospheres showed substantial structural integrity reinforcement during electrochemical reactions with improved sodium storage properties. Furthermore, there was high reversible capacity due to a large number of active sites, ideal void space and porosity for volume expansion, high surface permeability, and favorable kinetics due to the high face-to-volume ratio of the hollow structure. 

Nowadays, 3D composites of SnS_2_/Carbon architectures are becoming an academic hotspot with optimal rate capability and cycling stability owing to the synergism of active SnS_2_ particles and an extremely conductive carbon framework. Three-dimensional carbon fiber and graphene foam have served as a conductive and robust skeleton for SnS_2_, and their TEM imaging demonstrated that the SnS_2_ nanoflakes were strongly attached to these materials [172,173]. The graphene-assembled architectures can adapt hierarchical morphology with high surface-area-to-volume ratios and construct macroscopic and large-size monolithic materials, indicating that they have considerable technological promise for a variety of sustainable applications [174].

## 4. Applications of Synthesized SnS_2_ and SnS_2_/Carbon Nanomaterials in Environmental Remediation, Electrochemical Energy Conversion, and Storage

**Figure 14:**
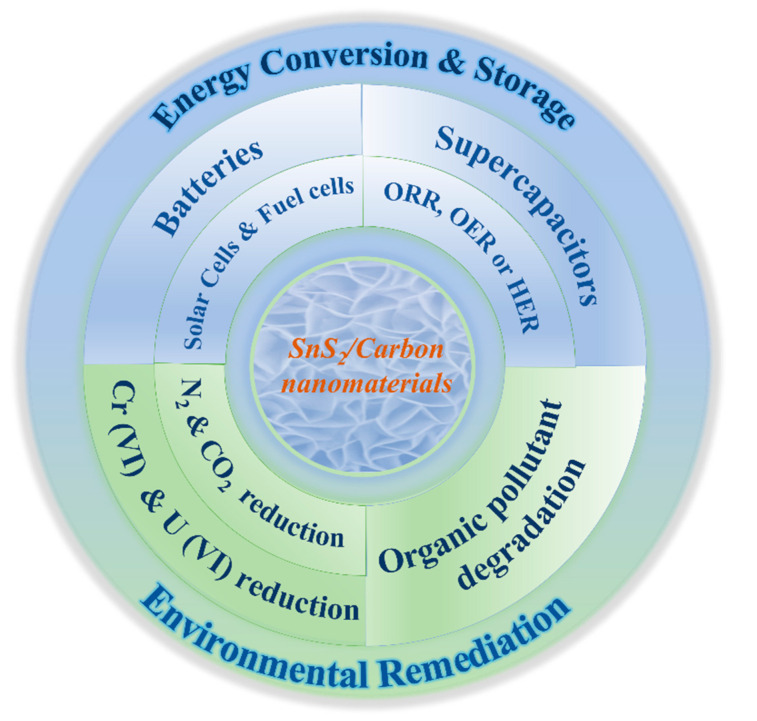
Applications of SnS_2_ and SnS_2_/Carbon nanomaterials in environmental remediation, electrochemical energy conversion, and storage.

Due to its extensive availability, biocompatibility, cheap cost, low toxicity, and high chemical stability, SnS_2_ is one of the most economically viable materials exploited in a wide range of applications. In addition, SnS_2_ possesses good qualities such as a high surface area with increased active sites, good ion exchange capability, and loading capacity. The hybridization of SnS_2_ with carbon materials has been explored in catalysis, biomedicine, supercapacitors, electrochemical sensors, batteries, photocatalysis, and so on. In particular, their capacity to build dimensionally variable structures gives SnS_2_ and SnS_2_/Carbon nanomaterials significant structural advantages in environmental remediation and electrochemical energy conversion and storage. The applications of SnS_2_ and SnS_2_/Carbon nanomaterials have been briefly summarized in Figure 14.

### 4.1. Photocatalyst in Pollutant Degradation

Photocatalysis has shown great potential in hydrogen production, antibacterial activity, pollutant degradation, air purification, etc. [175,176,177,178,179]. Amongst them, photocatalytic pollutant degradation is a particularly appealing technology since organic pollutants can be entirely degraded into CO_2_, H_2_O, and inorganic compounds leaving minimum detrimental leftovers [180,181]. For decades, semiconductor-based photocatalysts such as SnO_2_, ZnO, TiO_2_, etc., have gained prominence as breakthrough material for organic pollutant degradation [182]. This is due to their ability to use solar energy to carry out the catalytic reaction [183]. Amongst them, TiO_2_ has gained wider recognition due to its abundance and low cost. However, drawbacks of TiO_2_ such as a wide bandgap (3.2 eV), limited active sites, low absorption of UV light, and low quantum efficiency impede its versatility in the efficient degradation of pollutants [184]. Therefore, it is imperative to design a unique photocatalyst with high absorption capacity and a narrow bandgap for photocatalysis. SnS_2_ and its hybrid nanocomposites are gaining massive recognition in the scientific community as alternative photocatalytic materials to TiO_2_ as a result of their narrow bandgap and high quantum yield [185,186,187,188]. SnS_2_ composite nanomaterials have also shown higher catalytic performance than SnS_2_ nanomaterials themselves in pollutant removal.

#### 4.1.1. SnS_2_ Nanomaterials in Photocatalysis

SnS_2_ nanoparticles are known to exhibit photocatalytic properties under visible light [189,190,191]. As a semiconductor metal sulfide, SnS_2_ can act as capable sensitizers and harvest visible light for narrow bandgap semiconductors in some photocatalytic applications. Srinivas et al. found the bandgap of SnS_2_ nanostructures is around 2.50 eV as the photocatalyst of the irradiation of visible light [192]. SnS_2_ QDs have shown a bandgap that matches the absorption spectra of sunlight, a huge extinction coefficient due to quantum confinement, and large intrinsic dipole moments. However, the reduction in particle size has shown an increase in the bandgap of the semiconductor nanomaterials [193]. Nonetheless, various dimensions of SnS_2_ nanomaterials have reported successes in photocatalytic activities. For example, 1D SnS_2_ nanotubes have demonstrated big potential in photocatalysis with more active sites for adsorption and catalysis [194]. These properties have also been exhibited by 2D SnS_2_ nanomaterials [77,195]. For instance, atomically ultrathin 2D SnS_2_ conducting channels helped to achieve rapid carrier transport in photoelectrodes which greatly reduced the recombination rate with a bandgap of 2.29 eV [196]. Moreover, the lower thickness of 2D SnS_2_ structures provided an easy pathway for photogenerated electrons and holes to move toward the surface reaction sites [195]. Hence, the possibility of recombination is reduced, and photocatalytic effectiveness is improved. Ullah et al. in a comparative study observed that SnS_2_ and conventional cadmium sulfide (CdS) films have direct bandgap values of 2.20 eV and 2.45 eV, respectively [197].

Moreover, it was discovered that SnS_2_ film has a higher photocurrent of 140 µA than CdS films with 80 µA. Thus, compared with CdS, SnS_2_ nanostructures offer a better bandgap, superior cycling stability, and bigger reversible capacities that are desirable for photocatalysis and electrocatalytic applications. Three-dimensional SnS_2_ nanoflowers prepared at 120 °C in solvent ethylene glycol have been proven to have high adsorption capability and visible light photocatalytic activity for dyes (Methyl Blue and Methyl Orange) and heavy metal ions (Pb^2+^ and Cd^2+^) [198]. Microwave-assisted synthesis of hexagonal SnS_2_ allowed for the simultaneous adjustment of morphologies and nanostructures under atmospheric pressure and low temperature [199]. Moreover, it showed advantages in the photoreduction of stable azo-dye. In addition, SnS_2_ nanostructures showed excellent photocatalytic activity in the reduction of hazardous Cr(VI) to harmless Cr(III) in environmental conditions, as well as effectively decomposing mutagenic dyes (Methyl Orange and Rhodamine Blue) to benign compounds in a brief duration [192].

It is apparent that all dimensional SnS_2_ nanomaterials can be harnessed for photocatalytic applications due to their semiconductor nature. Researchers are drawn to this exceptional catalytic feat because it allows them to include and synthesize composite nanostructures with improved performance. Moreover, the bandgap is an important parameter in photocatalytic activities. Besides adsorption capacities, semiconductor catalysts with a narrow bandgap can absorb more photons, resulting in better catalytic activity when exposed to visible light [200]. New modifications are being used during synthesis to further optimize the bandgap for visible light application. One such modification is the introduction of carbon precursors to achieve SnS_2_/Carbon composite nanomaterials with desired morphological orientations and photocatalytic properties.

#### 4.1.2. SnS_2_/Carbon Nanomaterials in Photocatalysis

**Figure 15:**
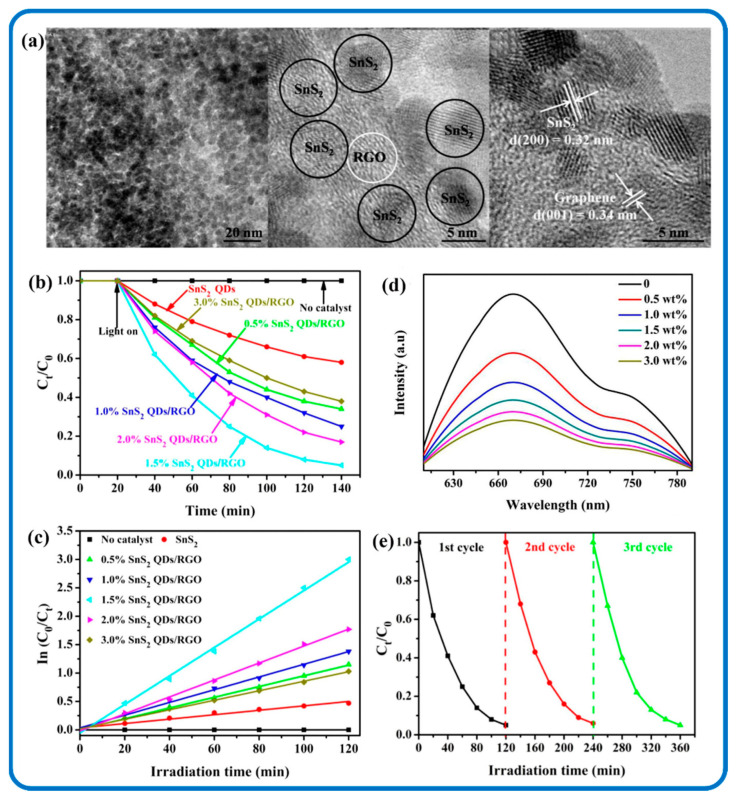
SnS_2_ QDs/rGO nanocomposite photocatalyst (**a**) TEM and HRTEM images, (**b**) Cr (VI) reduction efficiency by photocatalysis, (**c**) kinetic linear simulation cures of Cr(VI) degradation, (**d**) UV–*vis* absorption spectra of SnS_2_ loaded with different amounts of rGO, and (**e**) cycling runs of the photoreduction of Cr(VI) in the presence of SnS_2_ QDs/rGO photocatalyst. Adapted with permission from [206]. Copyright 2016, Elsevier.

Carbon materials can form unique chemical bonding thus providing strong interactions with SnS_2_, which leads to a bandgap narrowing effect [201]. SnS_2_/Carbon composite nanomaterials show more active sites, electron acceptors, and transport channels with improved structural stability and adsorption ability [202]. SnS_2_/Carbon nanomaterials have been reported to have the ability to degrade organic pollutants and carcinogens more effectively as compared to SnS_2_ (i.e., CO_2_) [103,203]. Xue et al. in their research used heterojunction bio-carbon/SnS_2_ nanocomposites with a narrow bandgap to efficiently photocatalyze the conversion of Arsenic(III) and calcium arsenate removal [204]. The -C=Sn-S bonds efficiently prevented SnS_2_ agglomeration, extended the photoresponse range, and enhanced the hydrophilicity of the bio-carbon/SnS_2_ nanocomposites while reducing their transfer resistance. For example, Figure 15 shows the sheet-like SnS_2_ nanoparticles uniformly incorporated on rGO sheets. Because of the increase in interfacial charge carriers, the addition of rGO to the composite nanomaterials improved the photocatalytic activity of Cr (VI) reduction. The SnS_2_/rGO composite photocatalysts also outperformed pure SnS_2_ QDs in terms of photocatalysis. So, the synergy between SnS_2_ and carbon materials at the nanoscale can provide a sufficient bandgap to catalyze photocatalytic reactions. A substantial bandgap is necessary to significantly promote the photocatalytic abilities of SnS_2_/Carbon composite nanomaterials [205].

**Figure 16:**
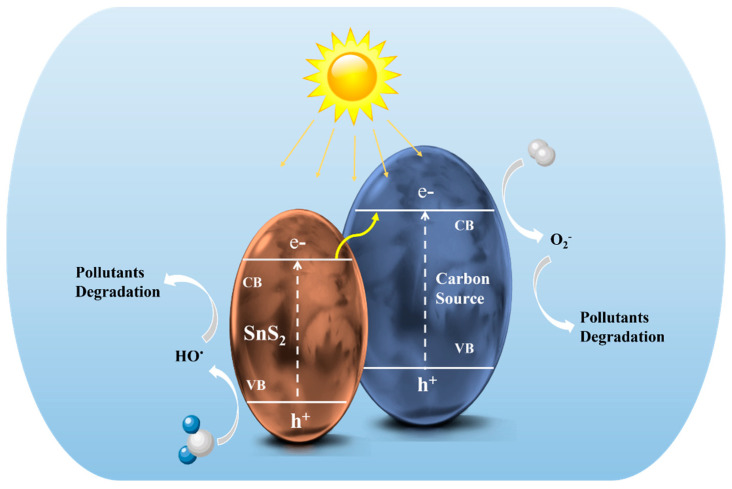
Photocatalytic schematic representation of SnS_2_/Carbon nanomaterials.

The recombination inhibition of charge carriers between SnS_2_ and the carbon materials has been observed to bring about the optimization of charge carriers at the SnS_2_/carbon interfaces to photodegrade Cr(VI) [207]. This was achieved through the coupling effect and the strong electrostatic attraction of carbon materials, which served as the electron acceptor to trap the photoinduced electrons from SnS_2_ and thus enhances the separation efficiency of electrons and holes [208]. However, the degradation of toxic substances is further impacted by the concentration of the pollutants and the dosage of the catalysts. Figure 16 further shows a schematic illustration of the mechanism in the photocatalytic breakdown of organic and inorganic pollutants by SnS_2_/Carbon composite nanomaterials. As shown in the diagram, once illuminated by light, electrons get excited and then migrate from the valence band (VB) to the conductor band (CB) of SnS_2_ QDs. Subsequently, the SnS_2_ electrons transfer to the associated carbon nanostructures that act as electron acceptors. This suppresses the recombination of photogenerated electron–hole pairs leading to ˙OH and ˙O^2−^ radical species, which can lead to the removal of pollutants by their superior activities. 

Table 3 shows the comparative photocatalytic performances of SnS_2_ and SnS_2_/Carbon composite nanomaterials under visible light from various literature. It can be observed that the synergistic combination of SnS_2_ nanomaterials and the carbon allotropes significantly enhanced the photocatalytic efficiency of the SnS_2_/Carbon composite nanomaterials compared with SnS_2_. Notably, dimensional SnS_2_/Carbon nanomaterials exhibited remarkable degrading effects especially on chromium (VI). Overall, SnS_2_/Carbon nanomaterials hold great degradation potential toward wastewater treatments. Photocatalysts are usually made of costly precious metals that are not in abundance. With the availability and low cost of SnS_2_ and carbon materials, researchers can venture more into creating SnS_2_/Carbon photocatalytic nanomaterials to harness its potential in photocatalysis at a large scale.

### 4.2. Electrochemical Conversion and Energy Storage Applications of SnS_2_ and SnS_2_/Carbon Nanomaterials

The ever-growing demands for energy resources and environmental concerns have paved the way for the exploration and development of clean and sustainable energy alternatives. Electrochemical energy conversions and storage devices including supercapacitors, fuel cells, solar cells, and metal ions or air batteries have gained attention due to their environmentally benign nature and hold great potential as a fossil fuel replacement. Since its discovery in 2004, graphene has become one of the most promising materials in energy storage due to its remarkable electrochemical properties [212,213]. Furthermore, graphene has the tendency to form composite nanomaterials of different dimensions which helps to boost the overall catalytic and electrochemical performance. SnS_2_ possesses a theoretical capacity of ~1136 mAhg^−1^ [214] which is higher than that of graphene (744 mAh g^−1^), [215,216] making it valuable for battery application, solely or in a composite material. SnS_2_/Carbon (including various carbon allotropes) composite nanomaterials have also proven to be more efficient for energy storage systems because of their high conductivity, mechanical and thermal stability, and long cycle ability [217]. The relationship between SnS_2_ and SnS_2_/Carbon nanoarchitectures and their electrochemical performances are discussed below. 

#### 4.2.1. SnS_2_ Nanomaterials in Electrochemical Conversion and Energy Storage

SnS_2_ nanomaterials exhibit enhanced electrochemical performance due to their compact and consistent crystal structure with a reasonable thickness and crystallinity [218], which is also favorable for structural stability and quick ion transport during lithiation/delithiation processes. Various synthesis approaches are geared at improving the performance of SnS_2_ nanomaterials as alternative electrode materials. However, the capacity fading of SnS_2_ electrode materials persists due to significant volume changes during charging/discharging processes [219,220]. SnS_2_ nanostructures with different morphologies have been fabricated to resolve these challenges.

Studies on the use of low-dimensional SnS_2_ nanomaterials in electrochemical energy conversion and storage applications are scant, to the best of our knowledge. This is because SnS_2_ materials can be hindered by sluggish diffusion kinetics and an unavoidable volume change during discharging and charging processes. Nonetheless, a SnS_2_ nanowall electrode realized a high reversible capacity of 576 mAh g^−1^ at 500 mA g^−1^ and an excellent rate capability of ~370 mAh g^−1^ at 5 A g^−1^ in sodium ion batteries [221]. The sulfide matrix acts as a buffer to decrease the large strain caused by the volume expansion of tin nanostructures [222]. Unfortunately, in some cases, the large volume expansion induces aggregation of the Sn particles. As such, it can bring about the cracking, pulverization, and degradation of the electrode material which leads to capacity loss [223,224,225]. Nevertheless, flowerlike-SnS_2_ nanostructures with large specific surface areas and better average pore sizes have exhibited remarkable battery performance with excellent long-term cycling stability [49,226,227]. In addition, binders with superior dispersion and cohesiveness in electrodes have shown to improve the electrochemical performance of SnS_2_ as the anode for LIBs [228]. SnS_2_ monolayers boost Lithium mobility, although their adsorption strength is moderate compared to other nanostructures. Rolling the monolayer into a one-dimensional nanotube increases Lithium ions’ adsorption strength and diffusion rates [229].

In terms of air batteries, Khan et al. used 3D SnS_2_ nanopetals as an air electrode material for hybrid Na-air batteries. It displayed a low overpotential gap of 0.52 V, high round trip efficiency of 83%, high power density of 300 mW g^−1,^ and good rechargeability of up to 40 cycles [230]. Moreover, their electrocatalytic performance was linked to oxygen reduction reaction (ORR) and oxygen evolution reaction (OER). The hybrid cell charge potential (OER) is 3.57 V at the high current density of 20 mA g^−1^, which is comparable to the charge potential (3.47 V) of a hybrid cell with Platinum on Carbon (Pt/C), known to be the best catalyst for ORR at low current density (5 mA g^−1^) [226]. Chia et al. explored the prospects of SnS_2_ materials as alternative electrocatalysts in ORR, OER, and HER [227]. It was proven that SnS_2_ has high inherent electrocatalytic activity and a fast heterogeneous electron transfer (HET) rate. Moreover, Xia et al. recently used first-principle methods based on the density functional theory to study the electrocatalytic performance of transition metal atoms supported on a SnS_2_ monolayer [231]. The catalytic performance of SnS_2_ for OER and ORR was shown to be significantly enhanced by the surface of the SnS_2_ monolayer. There is limited literature on the use of SnS_2_ solely or as a composite electrocatalyst, but gaps created in this field could be harnessed to create high-performance bifunctional ORR, OER, and HER electrocatalysts in the future.

#### 4.2.2. SnS_2_/Carbon Nanomaterials in Electrochemical Conversion and Energy Storage

Carbon nanostructures have been demonstrated to have the ability to confine active materials in composite nanostructures. The addition of heteroatoms to carbon could increase its affinity for active materials, form a strong architecture, and speed up the electron and ion transfer process [173,232,233]. When associated with SnS_2_ nanostructures, SnS_2_/Carbon composite nanomaterials can tolerate the volume change and enhance the ion diffusion rate through porous structure construction; thus, it is valuable in resolving the rapid battery capacity fading [234,235,236]. Furthermore, it can enhance the weak interaction between non-polar carbon and polar polysulfides which reduces polysulfide leakage from carbon materials in lithium-sulfur batteries (LSBs) [156,157].

**Figure 17:**
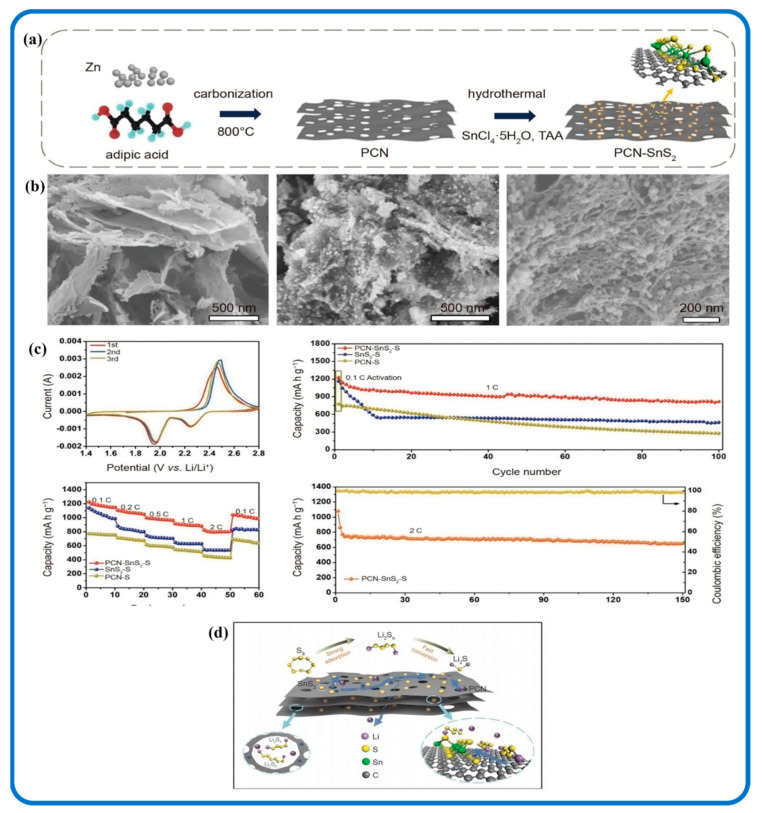
(**a**) Schematic preparation of PCN-SnS_2_ composites, (**b**) SEM images of porous carbon nanosheets (PCN) and PCN-SnS_2_, (**c**) overall lithium-sulfur battery performance of PCN-SnS_2_ nanocomposite, and (**d**) schematics of the conversion process of sulfur on PCNs-SnS_2_. Adapted with permission from Springer Nature [237]. Copyright 2021.

**Figure 18:**
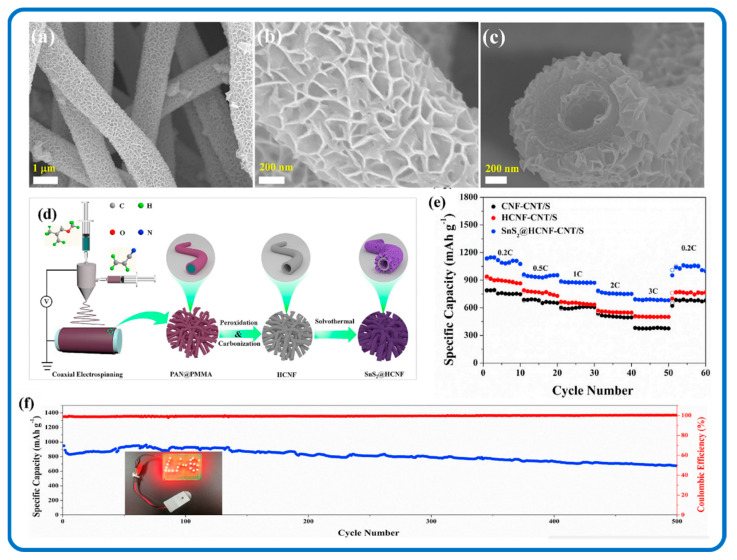
(**a**–**c**) SEM and TEM images, (**d**) schematic diagram of SnS_2_@HCNF synthesis, and (**e**,**f**) electrochemical performance of Li-S batteries with different interlayers. Reproduced with permission from [239]. Copyright 2021, Elsevier.

SnS_2_/Carbon nanomaterials as electrode materials in LSBs are fairly recent in research but have shown to have the ability to reduce the “shuttle effect”. Zhou et al. tried to resolve the limitations in LIBs by embedding SnS_2_ nanoparticles into 2D porous carbon nanosheet (PCN) interlayers to form a multi-functional (PCN-SnS_2_) nanocomposite as illustrated in Figure 17 [237]. The synergy between PCN and SnS_2_ nanoparticles resulted in a fast conversion of long-chain polysulfides to Li_2_S. The constant conversion of polysulfides on PCN- SnS_2_ to the final Li_2_S product assisted in reducing polysulfide shuttle during the cycling process. The best performance was demonstrated by PCN-SnS_2_ with dual physical-chemical confinement. It also improved the chemical reaction kinetics thereby diminishing the transfer of polysulfides to the lithium anode. This, in turn, reduced the “shuttle effect” during the entire charging/discharging process. Figure 17d shows a schematic illustration of the conversion process of sulfur on SnS_2_ embedded in PCNs. Wei et al. in Figure 18 also created a flexible electrocatalytic membrane that could reduce polysulfide shuttling and capacity fading in LSBs with different SnS_2_/HCNF (hollow carbon nanofiber) interlayers that are 2D nanostructured. The SnS_2_/HCNF in the LSBs displayed a high-rate discharge capacity (694 mAh g^−1^ at 3C) and low-capacity fading rate (0.056% per cycle during 500 cycles at 1C). Additionally, it showed that the nanocomposite efficiently alleviated the “shuttle effect” as a result of the composite nanostructure synergy [159,238].

**Figure 19:**
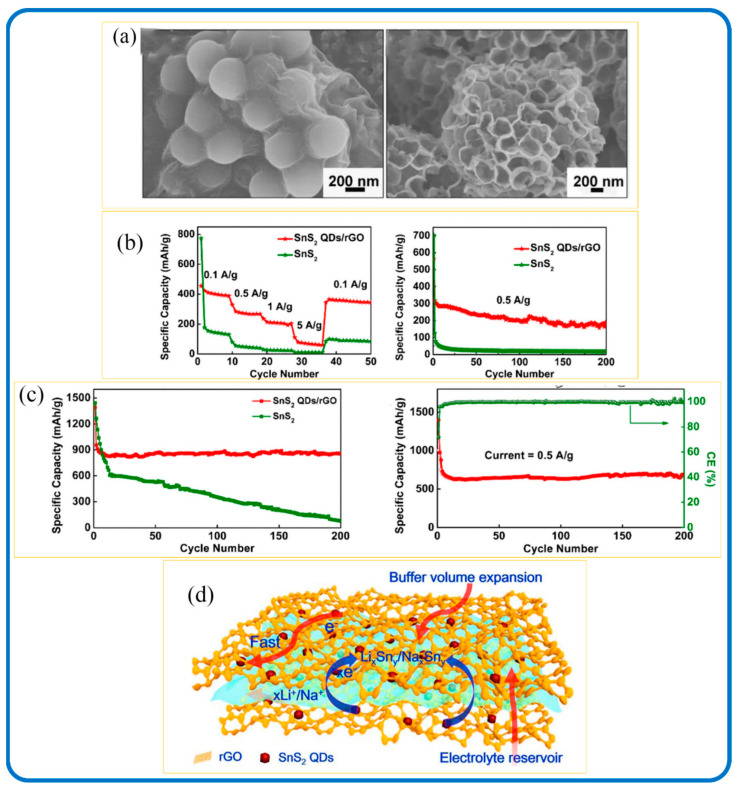
(**a**) SEM image, (**b**) schematic highlights during the charge/discharge processes of the 3D SnS_2_ QDs/rGO composite, (**c**) LIBs, (**d**) SIB performance of the SnS_2_ and 3D SnS_2_ QDs/rGO composite. Adapted from [243]. Copyright 2019, Springer, Open Access.

Three-dimensional nanomaterials are the most popular dimensional SnS_2_/Carbon composite nanostructures, and these nanostructures have also made an impact in energy storage applications. They have been beneficial for resolving the structure pulverization and poor electrical conductivity of metal dichalcogenides that could lead to adverse capacity decay both in LIBs and SIBs. Figure 19a,b shows the SEM image of 3D honeycomb-like rGO anchored with SnS_2_ quantum dots (3D SnS_2_ QDs/rGO) through spray-drying and sulfidation processes. The 3D features allowed for the volume change of SnS_2_ QDs during the lithiation/delithiation and sodiation/desodiation processes. It also made provision for electrolyte reservoirs to promote the conductivity of the SnS_2_ QDs. In addition, the 3D SnS_2_ QDs/rGO nanocomposite electrode delivered a high capacity and long cycling stability of 862 mAh g^−1^ for LIB at 0.1 A/g after 200 cycles (Figure 19c) and 233 mAh g^−1^ for SIB at 0.5 A g^−1^ after 200 cycles (Figure 19d). The improved battery performance, according to Chang et al., can be due to the composite structure’s robustness and the synergistic effects among a few layers of SnS_2_ and graphene [240]. Moreover, in situ-grown SnS_2_ nanoparticles have been homogeneously confined in rGO and CNT porous carbon nanostructures, which resulted in 3D architectures that demonstrated outstanding performance [55].

At present, the hybridization synthesis of SnS_2_/Carbon nanomaterials focuses on improving the capability and cycling stability of the electrodes [89,241]. To achieve a stable SIB/LIB electrode, Cui et al. developed self-standing electrodes with rational SnS_2_ nanosheets restricted into bubble-like carbon nanoreactors anchored on N, S doped carbon nanofibers [242]. The electrodes demonstrated a very steady capacity of 964.8 and 767.6 mAh g^−1^ at 0.2 A g^−1^, as well as strong capacity holding of 87.4% and 82.4% after 1000 cycles at high current density, respectively. It was stated further that the addition of N, S components improved the wettability of the carbon nanofiber matrix to the electrolyte and Li ions and the electrode’s overall electrical conductivity. The performances of SnS_2_ and SnS_2_/Carbon composite nanomaterials in battery applications are summarized in Table 4 and compared with graphene as a reference material. Numerous synthesis approaches are being harnessed to tackle these issues and formulate hybrid nanostructures with effective outcomes. This can perhaps shorten the pathway and improve the transportation speed of electrolyte ions at electrode surfaces [29].

Electrochemical reactions, such as ORR, OER, and hydrogen evolution reaction (HER) in fuel-cell and metal-air battery applications, have also shown promising successes in electrochemical energy conversion technologies [244,245,246,247]. However, research on SnS_2_/Carbon composite nanostructures as electrocatalysts is rarely reported. For instance, Cheng et al. fabricated stable SnS_2_ nanosheets incorporated with carbon dots, which exhibited an OER rate of up to 1.1 mmol g^−1^ h^−1^ under simulated sunlight irradiation [248]. Moreover, through a simple solid-state synthesis, a 2D SnS_2_/Graphene nanocomposite was achieved, and it showed an electrocatalytic (HER) overpotential of 0.36 V and a specific capacitance of 565 F g^−1^ [141]. In addition, a 3D hollow C@SnS_2_/SnS nanosphere was discovered to have outstanding OER performance through structural phase transitions [145]. The Sn^4+^ in the composite readily received electrons in water which is vital for improving the OER activity. More so, it measured a low overpotential of 380 mV at 10 mA cm^−2^ current density. Additionally, Chen et al. recently engineered SnS_2_ nanosheet arrays on carbon paper with surface oxygen adjustment under the directions of density function theory (DFT) calculations to efficiently electroreduce CO_2_ into formate and syngas (CO and H_2_) [249]. The SnS_2_ nanosheets that were modified with surface oxygen exhibited a notable Faradaic efficiency of 91.6% for carbonaceous products at −0.9 V vs. reversible hydrogen electrode (RHE), including 83.2% for formate creation and 16.5% for syngas. These dimensional SnS_2_ and SnS_2_/Carbon composite nanostructures can shorten electron transfer channels in electrochemical application because of their high surface-to-volume ratio, which probably have promoted their electrochemical performance.

**Table 4:** Comparison of battery performances of SnS_2_ and SnS_2_/Carbon composite nanomaterials.

Dimension	Materials	High Reversible Capacity (mAh g^−1^)	Cycle	Capacity Retention	Applications	Ref.
1D	SnS_2_	-	-	-	-	-
	SnS_2_/Carbon Nanotubes	940 & 605	200	91.2% & 87.6% @100 mA/g	LIB/SIB	[55]
	SnS_2_/Carbon Nanotubes	513.8	10	82% @100 mA/g	LIBs	[58]
	Polypyrrole/SnS_2_/Carbon	1009	100	97.7% @100 mA/g	LIBs	[93]
	SnS_2_/Graphene Nanorods	335	350	92% @100 mA/g	LIBs	[135]
	SnS_2_/HCNF ^1^	675	500	92.3% @ 100 mA/g	LSBs	[239]
	SnS_2_/Carbon (MWNTs) ^2^	768	100	78% @ 100 mA/g	SIBs	[250]
	SnS_2_/Carbon Nanofibers	457	~1000@2 A/g	89.5% @ 50 mA/g	PIBs ^5^	[251]
2D	SnS_2_ Nanosheets	733	50	100 mA/g	SIB	[125]
	SnS_2_ Nanoplates	521	50	90% @ 100 mA/g	LIBs	[218]
	SnS_2_/PCN ^3^	816	100	-	LSBs	[237]
	SnS_2_/EPC ^4^	443	450	89.4% @100 mA/g	SIBs	[252]
	SnS_2_/Graphene	911	200	89% @ 100 mA/g	LIBs	[253]
	SnS_2_/rGO	738	60	76.5% @ 0.2 C	LIBs	[254]
3D	SnS_2_ Nanoflowers	557	50	65% @ 0.1 C	LIBs	[53]
	SnS_2_ Nanoflowers	549.5	10	73% @ 100 mA/g	LIBs	[129]
	SnS_2_ Nanoflowers	502	50	84% @ 0.3 C	LIBs	[255]
	SnS_2_/Carbon	960	300	95% @ 100 mA/g	LIBs	[256]
	SnS_2_/Carbon-rGO	953	90	100 mA/g	LIBs	[257]
	SnS_2_/Carbon Nanoflowers	551	50	97% @ 100 mA/g	LIBs	[148]
	SnS_2_/Carbon Nanocubes	1080.1	200	84.1% @ 100 mA/g	LIBs	[109]
	SnS_2_/Carbon Nanospheres	690	150 @ 1 A/g	87% @ 100 mA/g	SIBS	[110]

^1^ HCNF, Hollow carbon nanofibers, ^2^ MWNTs, Multi-walled carbon, ^3^ PCN, Porous carbon nanosheet, ^4^ EPC, Enteromorpha Prolifera-derived carbon, ^5^ PIBs, Potassium-ion batteries.

## 5. Conclusions and Perspectives

SnS_2_ nanomaterials of different dimensional morphological orientations have made ample progress in photocatalysis and energy storage batteries. Meanwhile, they have presented some limitations which need further modifications to enhance their practical application potential. The broad bandgap and volume expansion during the charging/discharging processes of SnS_2_ are well-known drawbacks that limit its applicability. Hybridization of SnS_2_ with appropriate carbon materials, synthesizing composite nanomaterials, and developing innovative structures or morphologies dimensionally have been developed in order to overcome the aforementioned difficulties. Many novel and cost-effective synthetic methodologies have offered ways to achieve better performance in photocatalysis and energy storage batteries. SnS_2_/Carbon architectural nanomaterials have become an academic hotspot with outstanding reports on rate capability and cycling stability due to the synergism of active SnS_2_ particles and a very conductive carbon framework.

Here, we have summed up some recent research on SnS_2_ and SnS_2_/Carbon composite nanomaterials and reviewed the progress made on the wet and solid-phase fabrication methods to achieve various morphological structures of tin disulfide (SnS_2_) and SnS_2_/Carbon nanomaterials such as nanodots, nanofibers, nanowires, nanotubes, nanorods, nanosheets, nanoflowers, and nanospheres in (0D–3D) dimensional states and their applications in photocatalysis, electrochemical conversion, and energy storage. We tried to bridge the knowledge gap presented in SnS_2_, SnS_2_/Carbon nanostructures, and their application performances in photocatalytic degradation and energy storage batteries. 

Although the understanding of dimensional hybrid nanomaterials has made some achievements, there is still room to harness their dimensional capabilities, as this field of study has a lot of promise for the development of high-performance nanomaterials. In the meantime, more research into the compatibility of carbon nanomaterials with SnS_2_ functional nanomaterials is needed to enhance the utilization of these hybrid nanocomposites in photocatalytic and energy storage applications. Furthermore, a deeper knowledge of the mechanisms involved in the formation of SnS_2_/Carbon nanohybrids can be used to develop novel methods for producing optimal, cost-effective, and environmentally benign composite nanomaterials. Realizing these possibilities may necessitate the efforts of researchers as well as a fresh look at hierarchical nanocomposites.

## Figures and Tables

**Table 1:** Comparison of the various SnS_2_/Carbon nanomaterials achieved through wet and solid-state synthesis approaches and their applications.

Dimension	Synthesis Method	SnS_2_/Carbon Composites	Applications	Ref.
0D	Solid-State Synthesis	SnS_2_/Fullerene	-	[91]
1D	Facile Electrospinning Technique	SnS_2_/ NSDC ^1^ Nanofibers	SIBs ^2^	[92]
	Hydrothermal Method	Polypyrrole@SnS_2_@Carbon Nanofiber	LIBs ^3^	[93]
	Facile Sintering Route	SnS_2_ Cross-Linked/ CNTs ^4^	SIBs	[94]
	Solvothermal Method	SnS_2_ Nanoflakes/CNT	LIBs	[95]
	Hydrothermal Method	SnS_2_@rGF ^5^	SIBs	[96]
	Plasma Evaporation and Post Sulfurization	SnS_2_ Semi-Filled CNT	LIBs	[97]
2D	Hydrothermal Method	SnS_2_/rGO ^6^	LIBs	[98]
	Hydrothermal Method	SnS_2_/Graphene Aerogel	SIBs	[99]
	Hydrothermal Method	SnS_2_/Graphene	SIBs	[100]
	Thermal Annealing	SnS_2_/rGO	SIBs	[101]
	Ultrasonication	SnS_2_/Graphene	LIBs/ SIBs	[102]
	Hydrothermal Method	Carbon-Doped SnS_2_	CO_2_ Reduction in Fuel Cell	[103]
	Solvothermal Method	SnO_2_-rGO/SnS_2_	NO_2_ detection	[104]
	Thermal Annealing	SnS_2_/N-Doped rGO	LIBs	[105]
	Wet Chemical Transfer Method	Graphene/SnS_2_ Heterojunction	Photoelectric Performance	[106]
3D	Solvothermal Method	SnS_2_/GO Nanoflower	Ultrasensitive Humidity Sensor	[107]
	Hydrothermal Method	SnS_2_/Graphene Monolith	SIBs	[108]
	Thermally Annealing	SnS_2_/N-Doped Cubic-Like Carbon	LIBs	[109]
	Solvothermal Method	SnS_2_/Carbon Yolk-Shell	SIBs	[110]
	Hydrothermal Method	SnS_2_ Flowers/Carbon Nanotubes	SIBs	[111]
	Hydrothermal Method	SnS_2_@Carbon Hollow Nanospheres	SIBs	[112]
	Hydrothermal Method	SnS_2_/rGO Spheres	Asymmetric Supercapacitors	[113]
	Hydrothermal Method	SnS_2_/g-C_3_N_4_ ^7^ Amorphous Spheres	Supercapacitors	[114]

^1^ NSDC, Nitrogen, Sulfur-doped carbon nanofibers; ^2^ SIBs, Sodium ion batteries; ^3^ LIBs, Lithium ion batteries; ^4^ CNT, Carbon nanotube; ^5^ GF, Graphene fiber; ^6^ rGO, Reduced graphene oxide; ^7^ g-C_3_N_4_, graphitic carbon nitride.

**Table 3:** Comparison of the photocatalytic activity of SnS_2_ and SnS_2_/Carbon nanomaterials on pollutant remediation.

Dimension	Photocatalysts	Pollutants	Photocatalytic Efficiency (%)	Irradiation Time (min)	Ref.
0D	SnS_2_ Quantum Dots	Chromium (VI)	92	120	[118]
	SnS_2_ Nanoparticles	Methyl Orange	90	60	[199]
	SnS_2_ QDs/rGO	Chromium (VI)	95.3	120	[206]
	SnS_2_ QDs/N-doped Graphene	Methyl Orange	95.6	60	[134]
1D	SnS_2_ Nanotubes	Chromium (VI)	53.0	60	[194]
	CNT@MoS_2_/SnS_2_	Chromium (VI)	~100	90	[202]
2D	SnS_2_ Nanoflakes	Rhodamine B	61	120	[44]
	SnS_2_ Nanoflakes	RR 120 Dye	-	180	[78]
	SnS_2_ Nanoplates	Methyl Blue	85	120	[195]
	SnS_2_/rGO	Chromium (VI)	94.0	90	[60]
	Bio-carbon/SnS_2_ Nanosheets	Arsenic (III)	95.1	-	[204]
	SnS_2_/N-Doped Carbon QDs	Chromium (VI)	100	25	[208]
	SnS_2_-SnO_2_/Graphene	Rhodamine Blue	97.1	60	[209]
3D	SnS_2_ Nanoflowers	Chromium (VI)	83.8	-	[27]
	SnS_2_ Nanoflowers	Methyl Orange	79.8	120	[198]
	Carbon Dot-SnS_2_	Chromium (VI)	77.3	-	[207]
	SnS_2_/rGO	Chromium (VI)	90.0	150	[210]
	Carbon/SnS_2_	Chromium (VI)	99.7	120	[211]

## Data Availability

Not applicable.
